# Experiences of patients with Poland syndrome of diagnosis and care in Italy: a pilot survey

**DOI:** 10.1186/s13023-019-1253-8

**Published:** 2019-11-21

**Authors:** Ilaria Baldelli, Fabio Gallo, Marco Crimi, Piero Fregatti, Lorenzo Mellini, Pierluigi Santi, Rosagemma Ciliberti

**Affiliations:** 1 Department of Surgical Sciences and Integrated Diagnostics (DISC), University of Genoa, Genoa, Italy; 2 Policlinico San Martino Hospital IRCCS for Oncology, L.go R. Benzi 10, 16132 Genoa, Italy; 3 Executive Committee Italian Association of Poland Syndrome (AISP), Genoa, Italy; 4 Scientific Committee Italian Association of Poland Syndrome (AISP), Genoa, Italy; 5 Department of Health Science (DISSAL), University of Genoa, Genoa, Italy; 6 Kaleidos SCS-Onlus, Scientific Office, Bergamo, Italy

**Keywords:** Poland syndrome, Breast asymmetry, Chest malformation, Hand malformation, Congenital malformation, Rare disease, Underdiagnosed patients

## Abstract

**Background:**

Poland Syndrome (PS) is a rare congenital malformation involving functional and aesthetic impairments. Early diagnosis and timely therapeutic approaches play an important role in improving the quality of life of patients and kindred. This study aims to explore healthcare experiences of the diagnosis of patients affected by PS and to investigate the factors associated with diagnostic delay in Italy.

**Results:**

Seventy-two patients affected by PS were asked to fill in a self- administered questionnaire on: a) diagnostic path; b) perceived quality of care received after diagnosis; c) knowledge of the rights and the socio-economic hardships related to their disease; d) evaluation of the integration of various professional skills involved in the diagnostic and therapeutic approach; e) perception of the social support provided by the Italian Association of Poland Syndrome (AISP). The average age at diagnosis was around 14 years; diagnosis was made at birth in only 31.58% of cases. Although typical symptomatology had appeared on average at an early age (4 months), only 23 patients (40.35%) received an early diagnosis (within the first year of life). Just over half of the patients (*n* = 30) were diagnosed in their region of origin, while 27 were diagnosed elsewhere. Furthermore, 12.28% were self-diagnoses. Among the patients who were diagnosed outside their region, 15 (88.24%) stated they had foregone some visits or treatments owing to costs and/or organizational issues.

**Conclusions:**

An analysis of the patients’ experiences highlights several gaps and a lack of homogeneity in the diagnostic and therapeutic follow-up of PS patients in Italy. A specific national diagnostic and therapeutic path is essential to guarantee patients complete and appropriate health services, compliant with the ethical principles of non-discrimination, justice and empathy. Implementation of an effective information and research network and empowerment of patients’ associations are necessary conditions to encourage clinical collaboration and improve the quality of life of people living with rare diseases.

## Background

Poland Syndrome (PS) is a rare congenital condition characterized by unilateral, partial or complete lack of the pectoralis major. Various rib cage deformities, ipsilateral hand anomalies and ipsilateral breast hypoplasia or agenesis may be observed [1–3]. According to the National Human Genome Research Institute, PS affects males three times more often than females and affects the right side of the body twice as often as the left [4]. The incidence ranges from one out of 7000 to one out of 100,000 live births [5]. Despite various hypotheses, the pathogenic mechanism underlying PS is still unknown [6, 7]. So, despite the greater access to comprehensive genetic testing, patients with PS, as individuals with only suspected genetic condition, often undertake the “diagnostic odyssey” characterized by years of clinical, radiological, genetic and laboratory testing. During their diagnostic odyssey, emotional distress and frustration in parents and adult patients have been observed [8–11]. The parents of patients without a diagnosis feel uncertain about the future of their children because they lack information on how to manage the symptoms that arise. On the other hand, adult patients seeking a diagnosis hope to be able to improve their quality of life. The diagnosis, in fact, can be considered a starting point towards treatment options, thus reducing uncertainty [12]. Moreover, receiving a correct diagnosis and benefiting from appropriate genetic counseling is crucial for planning future pregnancies, both for parents and patients, through an informed choice about their reproductive options [13]. Early diagnosis also plays an important role in scheduling a timely therapeutic approach. Patients affected by PS, for example, need to correct hand deformities [14] during childhood to reduce functional impairment. Severe chest/breast asymmetries [2] need to be addressed during adolescence to avoid negative body perception which may lead to permanent insecurities and the development of body-image disorders [15].

To date, in Italy, PS is often unrecognized, and only two regions, both of which are in Northern Italy (Liguria and Lombardy), have formally published a specific Diagnostic and Therapeutic Care Paths (PDTA) and are able to offer patients complete and appropriate health services [16, 17]. There is little or no information on how well the healthcare needs of people living with PS in Italy are being met. Thus, the first aim of this study was to explore the experiences of a group of Italian patients affected by PS with regard to diagnosis and care. The second aim was to investigate the factors associated with diagnostic delay in Italy.

## Methods

The study involved 72 PS patients from various Italian regions, who underwent examination (either their first or for follow-up) during two national meetings focused on patients affected by PS, which were held in Genova in 2016. While in the waiting room, adult patients (≥ 18 yo) and caregivers of underage patients were asked to fill in an anonymous, voluntary, self-administered, multiple-choice questionnaire (Table 1). The final version was composed of 26 questions (83 items) and was divided into “demographic information” (age, sex, region of origin, respondent) and 5 other sections: a) the first section “Diagnostic Path” referred to the timing of both the appearance of symptoms and confirmation of the diagnosis, to the healthcare facility and to the region where the diagnosis was confirmed; b) the second section “Quality of Care after Diagnosis” inquired about the respondents’ degree of difficulty in identifying a specialist for treatment, the perceived quality of healthcare services for PS, the impact of the difficulties they encountered, the presence of a reference healthcare provider during the treatment period and the feeling of being understood; c) the third section “Rights and Socio-economic Hardships encountered” asked respondents to indicate if and how they became aware of their health rights, if they took advantage of health-cost exemption and if it was sufficient, if they felt protected by the Italian NHS and if they had been forced to forgo some treatments; d) the fourth section “Collaboration among Health Care Professionals” asked respondents to rate the involvement of pediatricians, general practitioners and specialists in the diagnostic and therapeutic approach; e) the fifth section “Associative Support and Activities” investigated the perception of the usefulness of a patients’ association and the role of the Italian Association of Poland Syndrome (AISP) throughout the patients’ care path, their own personal registration and participation in association activities and, finally, the usefulness of medical meeting days dedicated to PS; f) the last section “Scientific Research” investigated the patients’ opinions on the scientific research activities promoted by the association.

**Table 1:** Questions from the questionnaire, divided by topic group

A) DIAGNOSTIC PATH	
• Time of Diagnosis: when was the diagnosis confirmed?	
• Who Diagnosed: who made the diagnosis?	
• Region of Diagnosis: was the diagnosis made in your home region?	
• Time of Symptoms Onset: at what age did the first signs of Poland syndrome appeare?	
B) QUALITY OF CARE AFTER DIAGNOSIS	
• Difficulty to Find a Specialist for Treatment: Did you find it difficult to identify a specialist to treat your condition?	
• Quality of Treatment: How do you feel that your disease is being treated?	
• Impact: What impact did the difficulties you encountered in the treatment course have on you?	
• Reference Figure during Treatment: Did you know who to refer to during the course of treatment?	
• Feeling Understood during Treatment: Did you feel understood by the doctor during the communication process?	
C) RIGHTS AND SOCIO-ECONOMIC HARDSHIP ENCOUNTERED	
• Knowledge of tax Exemption**:** Are you aware of the possibility of accessing health benefits in relation to your condition?	
• How Found Out About Exemption: How did you access the benefits of exemption and / or disease monitoring?	
• Exemption: have you been given an exemption for your condition?	
• Sufficiency of the Exemption: Does the exemption fully cover the diagnostic and therapeutic needs of your condition?	
• Feeling of Protection: do you feel sufficiently protected by the health care structure of your region?	
• Forgo Treatment: have you ever forgone some visits or treatments because of the costs or organizational issues you encountered?	
D) COLLABORATION AMONG HEALTH CARE PROFESSIONALS	
• General Practitioners _Pediatrician Role: How was the relationship with your general practitioner / pediatrician during the diagnostic and therapeutic clinical course? • Collaboration: did your family doctor and specialist collaborate with each other?	
E) ASSOCIATIVE SUPPORT AND ACTIVITIES	
• AISP Usefulness: Do you think a patient’s association is useful for your pathology?	
• AISP Advantage in care: Do you think that AISP has somehow facilitated your treatment path?	
• Join AISP: Are you enrolled in the Italian association of Poland syndrome?	
• Participate in AISP Activities: do you participate in the activities of the association?	
• PolandDay Usefulness: Do you think AISP promotes your relationship with the various health services and operators?	
F) SCIENTIFIC RESEARCH	
• Volunteer: Would you like to contribute as a volunteer, even occasionally, to the activities of the Association?	
• Research: Do you think that scientific research is important in finding the cause of Poland syndrome?	
• GeneticDonation: Would you donate your genetic material for research?	
• Family involvement in genetic research: Would you share the results of genetic tests and research with your family members?	

The questionnaire was designed by a panel of experts on PS who were members of either the scientific committee (clinicians) or of the executive committee (patients or parents of affected patients who are volunteers of the association) of AISP. The questionnaire was specifically designed to detect the difficulties encountered by patients suffering from PS in the diagnostic and therapeutic care path in Italy and thus to develop targeted actions. Clinicians, supported by a bioethicist, drew up a draft of the survey composed of 41 questions and 150 items. The other experts (patients or parents of affected patients) were asked to rate each question and each item of the survey by assigning a score of 0, 1, 2 or 3 (0 = not pertinent and not explicit; 1 = not pertinent but explicit; 2 = pertinent but not explicit; 3 = pertinent and explicit). Those that were rated as “0” or “1” were removed, while those rated “2” were modified. In the end, 15 questions were removed and 8 items were modified. Given the originally envisioned internal use for which the questionnaire was created, no other validation or reliability methods were applied.

### Statistical analysis

Continuous variables are presented as means with standard deviations (SD), and categorical variables as numbers of subjects and percentage values. If patients received the diagnosis of PS in the region where they lived, the region was classified as “IN” otherwise as “OUT”. The diagnosis time was classified as “*EARLY*” (PS diagnosis within the first year of life) or “*LATE*” (PS diagnosis after the first year of life). Moreover, the region where PS patients lived was grouped into “Liguria, Lombardia”, where an official PDTA for PS is present, or “Others”.

Univariate Penalized Logistic Regression models were implemented in order to screen the effect of clinical and demographic variables on the region of diagnosis and on the diagnosis time. The odds ratio associated with the region of diagnosis, and their 95% confidence intervals, were calculated for each factor from the Logistic model. The Likelihood Ratio (LR) test was used to test statistical significance. Covariates with a *p*-value < 0.05 were then selected for multivariate analysis, in which the region of diagnosis and the diagnosis time were the dependent variables. Multivariate analysis was also performed by the Penalized Logistic Regression model, and model selection was carried out using the Akaike Information Criterion (AIC). Differences with a *p*-value < 0.05 were deemed significant, and data were acquired and analyzed in the R v3.5.1 software environment [18].

## Results

The study involved 72 PS patients or caregivers who were asked to fill in a questionnaire, 57 (23 adults, 34 minors) of whom provided enough information to warrant evaluation. The demographic and clinical characteristics of the study participants are summarized in Table 2. Briefly, the mean age at the time of filling in the questionnaire was 24.52 ± 16.91 years; 25 patients (43.86%) were males and 32 (56.14%) were females. The patients were all born in Italy and came from 14 of the 20 Italian regions. Just over half of the patients (*N* = 30) received their PS diagnosis in their own region, while 27 had been diagnosed outside their region of origin. The average age at diagnosis was around 14 years; the diagnosis was made at birth in only 31.58% of cases. Although the typical symptomatology of the syndrome appeared on average at an early age (4 months), only 23 patients (40.35%) had received an early diagnosis (within the first year of life). Despite the difficulties encountered in obtaining a diagnosis, 45.61% of those interviewed rated the overall quality of care received after diagnosis as good, while 28.07% considered it excellent. However, almost half of the respondents reported having suffered some consequences because of the difficulties they encountered in the diagnostic path, two thirds of whom (35.09%) reported them as being of a psychological nature. Almost all the patients reported having been informed of the existence of a specific exemption from payment of the costs related to their pathology; however, 29.82% reported that they had waived health care on account of costs and/or organizational issues. The interviewees acknowledged the important role of AISP in facilitating the healthcare path (73.68%) and considered AISP to be useful (91.23%), while a marginal role (not significant = 38.6%; absent = 40.35%) was attributed to general practitioners.

**Table 2:** Demographic and clinical characteristics of study participants (*n* = 57). The results are expressed as mean with standard deviation or as number of subjects with percentage

Characteristic	Overall
Age when filling in the questionnaire	24.52 (16.91)
Gender	
MALE	25 (43.86%)
FAMALE	32 (56.14%)
Region Of Origin	
CALABRIA	1 (1.75%)
CAMPANIA	5 (8.77%)
EMILIA ROMAGNA	5 (8.77%)
LAZIO	6 (10.53%)
LIGURIA	7 (12.28%)
LOMBARDIA	7 (12.28%)
MOLISE	1 (1.75%)
PIEMONTE	4 (7.02%)
PUGLIA	5 (8.77%)
SARDEGNA	3 (5.26%)
SICILIA	3 (5.26%)
TOSCANA	6 (10.53%)
UMBRIA	1 (1.75%)
VENETO	3 (5.26%)
Time of Diagnosis	
EARLY	23 (40.35%)
LATE	34 (59.65%)
Who Diagnosed	
NEONATOLOGY UNIT	10 (17.54%)
PEDIATRICIAN	4 (7.02%)
GENERAL PRACTITIONER	1 (1.75%)
SPECIALIZED MED CENTRE	22 (38.6%)
SELF-DIAGNOSIS	7 (12.28%)
OTHER	13 (22.81%)
Region of Diagnosis	
IN	30 (52.63%)
OUT	27 (47.37%)
Time of Symptoms Onset	
AT BIRTH	52 (91.23%)
1 YEAR	1 (1.75%)
3 YEARS	2 (3.51%)
6 YEARS	1 (1.75%)
7 YEARS	1 (1.75%)
Difficulty Finding a Specialist for Treatment	
NO	29 (50.88%)
YES	26 (45.61%)
DON’T KNOW	2 (3.51%)
Quality of Treatment	
EXCELLENT	16 (28.07%)
GOOD	26 (45.61%)
SUFFICIENT	7 (12.28%)
UNSATISFACTORY	6 (10.53%)
OTHER	2 (3.51%)
Impact	
NOT MY CASE	24 (42.11%)
NONE	20 (35.09%)
IMPORTANT PSY IMPACT	11 (19.3%)
MILD PSY IMPACT	1 (1.75%)
FUNCTIONAL IMPAIRMENT	1 (1.75%)
OTHER	–
Reference Figure during Treatment	
NO	16 (28.07%)
YES	33 (57.89%)
DON’T KNOW	8 (14.04%)
Feeling Understood during Treatment	
NO	10 (17.54%)
YES	42 (73.68%)
DON’T KNOW	5 (8.77%)
Knowledge of tax Exemption	
NO	3 (5.26%)
YES	54 (94.74%)
How They Found Out about Exemption	
GENERAL PRACTITIONER	8 (14.81%)
REGIONAL BOOKING MEDICAL CENTER	–
AISP	28 (51.85%)
REFERENCE MEDICAL CENTER	14 (25.93%)
OTHER	4 (7.41%)
Exemption	
NO	11 (19.3%)
YES	46 (80.7%)
Sufficiency of the Exemption	
NO	12 (30.0%)
YES	28 (70.0%)
Feeling of Protection	
NO	27 (47.37%)
YES	12 (21.05%)
DON’T KNOW	18 (31.58%)
Forgo Treatment	
NO	33 (57.89%)
YES	17 (29.82%)
DON’T KNOW	7 (12.28%)
General Practitioner _Pediatrician’s Role	
FUNDAMENTAL	5 (8.77%)
QUITE RELEVANT	7 (12.28%)
NOT VERY RELEVANT	22 (38.6%)
NOT AT ALL	23 (40.35%)
Collaboration	
NO	40 (70.18%)
YES	6 (10.53%)
DON’T KNOW	11 (19.3%)
AISP Usefulness	
NO	1 (1.75%)
YES	52 (91.23%)
DON’T KNOW	4 (7.02%)
AISP Advantage in Care	
NO	3 (5.26%)
YES	42 (73.68%)
DON’T KNOW	12 (21.05%)
Join AISP	
NO	12 (21.05%)
YES	45 (78.95%)
Participate in AISP Activities	
NO	34 (59.65%)
YES	23 (40.35%)
PolandDay Usefulness	
NO	1 (1.75%)
PARTIAL	7 (12.28%)
YES	49 (85.96%)
Volunteer	
NO	7 (12.28%)
YES	19 (33.33%)
DON’T KNOW	31 (54.39%)
Research	
YES	56 (98.25%)
DON’T KNOW	1 (1.75%)
Genetic Donation	
YES	52 (91.23%)
DON’T KNOW	5 (8.77%)
Family Involvement in Genetic Research	
NO	4 (7.02%)
YES	43 (75.44%)
DON’T KNOW	10 (17.54%)

### Influence of the region of origin on diagnosis and care

Table 3 reports the statistic output of demographic and clinical factors related to the possibility of receiving the diagnosis in or out of the region of origin: among patients who were diagnosed outside their region, 15 (88.24%) stated they had forgone some visits or treatments owing to costs and/or organizational issues. The majority of PS patients (12 (85.71%)) that lived in Liguria or Lombardia received a PS diagnosis in a healthcare facility in their region, while 25 out of 43 (58.14%) patients who lived in the other Italian regions were diagnosed with PS outside their region of origin (*p* = 0.0041).

**Table 3:** Contingency tables and summary output of the univariate analysis. Characteristic: variable taken into account; OR (95% CI): Odds Ratio with 95% Confidence Interval; *p*-value: Likelihood Ratio *p*-value. ^a^Variables entering the multivariate analysis (see the text for abbreviations and further details)

Characteristic	Descriptive statistics		Univariate analysis	
	RegionDgn		OR (95%C.I.)	*p*-value
	IN	OUT		
Gender				0.0958
MALE	10 (40%)	15 (60%)	1	
FEMALE	20 (62.5%)	12 (37.5%)	0.41 (0.14: 1.17)	
Macro Region^a^				0.0041
LIGURIA, LOMBARDIA	12 (85.71%)	2 (14.29%)	1	
OTHERS	18 (41.86%)	25 (58.14%)	6.89 (1.78: 38.57)	
Time of Diagnosis^a^				0.0372
EARLY	16 (69.57%)	7 (30.43%)	1	
LATE	14 (41.18%)	20 (58.82%)	3.11 (1.07: 9.70)	
Who Diagnosed^a^				0.0045
NEONATOLOGY UNIT	9 (90%)	1 (10%)	1	
PEDIATRICIAN	3 (75%)	1 (25%)	2.71 (0.18: 43.60)	
GENERAL PRACTITIONER	0 (0%)	1 (100%)	19 (0.69: 3428.71)	
SPECIALIZED MED CENTRE	6 (27.27%)	16 (72.73%)	16.08 (2.84: 173.45)	
SELF-DIAGNOSIS	2 (28.57%)	5 (71.43%)	13.93 (1.73: 197.59)	
OTHER	10 (76.92%)	3 (23.08%)	2.11 (0.28: 24.78)	
Time of Symptoms Onset				0.9999
AT BIRTH	0 (NaN%)	0 (NaN%)	1	
LATER	3 (60%)	2 (40%)	1 (0.02: 60.68)	
Difficulty Finding a Specialist for Treatment				0.6585
NO	17 (58.62%)	12 (41.38%)	1	
YES	12 (46.15%)	14 (53.85%)	1.62 (0.57: 4.71)	
DON’T KNOW	1 (50%)	1 (50%)	1.4 (0.1: 18.82)	
Quality of Treatment				0.5953
EXCELLENT	8 (50%)	8 (50%)	1	
GOOD	16 (61.54%)	10 (38.46%)	0.64 (0.18: 2.17)	
SUFFICIENT	3 (42.86%)	4 (57.14%)	1.29 (0.24: 7.45)	
UNSATISFACTORY	3 (50%)	3 (50%)	1 (0.16: 6.08)	
OTHER	0 (0%)	2 (100%)	5 (0.34: 731.03)	
Impact				0.6293
NOT MY CASE	13 (54.17%)	11 (45.83%)	1	
NONE	8 (40%)	12 (60%)	1.73 (0.54: 5.73)	
IMPORTANT PSY IMPACT	7 (63.64%)	4 (36.36%)	0.70 (0.16: 2.84)	
MILD PSY IMPACT	1 (100%)	0 (0%)	0.39 (0.00: 8.09)	
FUNCTIONAL IMPAIRMENT	1 (100%)	0 (0%)	0.39 (0: 8.09)	
Reference Figure during Treatment				0.9440
NO	9 (56.25%)	7 (43.75%)	1	
YES	17 (51.52%)	16 (48.48%)	1.19 (0.37: 3.94)	
DON’T KNOW	4 (50%)	4 (50%)	1.27 (0.25: 6.63)	
Feeling Understood during Treatment				0.8257
NO	6 (60%)	4 (40%)	1	
YES	21 (50%)	21 (50%)	1.44 (0.38: 5.90)	
DON’T KNOW	3 (60%)	2 (40%)	1.03 (0.12: 7.95)	
Knowledge of tax Exemption				0.6776
NO	2 (66.67%)	1 (33.33%)	1	
YES	28 (51.85%)	26 (48.15%)	1.55 (0.19: 17.75)	
How They Found Out about Exemption				0.6893
GENERAL PRACTITIONER	5 (62.5%)	3 (37.5%)	1	
REGION BOOKING MEDICAL CENTER	14 (50%)	14 (50%)	1.57 (0.35: 7.92)	
AISP	6 (42.86%)	8 (57.14%)	2.05 (0.39: 12.06)	
REFERENCE MEDICAL CENTER	3 (75%)	1 (25%)	0.67 (0.05: 6.64)	
Exemption				0.8995
NO	6 (54.55%)	5 (45.45%)	1	
YES	24 (52.17%)	22 (47.83%)	1.09 (0.30: 4.02)	
Sufficiency of the Exemption				0.5038
NO	5 (41.67%)	7 (58.33%)	1	
YES	15 (53.57%)	13 (46.43%)	0.64 (0.16: 2.38)	
Feeling of Protection				0.0691
NO	10 (37.04%)	17 (62.96%)	1	
YES	9 (75%)	3 (25%)	0.22 (0.05: 0.88)	
DON’T KNOW	11 (61.11%)	7 (38.89%)	0.39 (0.11: 1.27)	
Forgo Treatment^a^				0.0003
NO	23 (69.7%)	10 (30.3%)	1	
YES	2 (11.76%)	15 (88.24%)	13.88 (3.47: 80.21)	
DON’T KNOW	5 (71.43%)	2 (28.57%)	1.02 (0.16: 5.08)	
General Practitioner _Pediatrician’s Role				0.3299
FUNDAMENTAL	2 (40%)	3 (60%)	1	
QUITE RELEVANT	6 (85.71%)	1 (14.29%)	0.16 (0.01: 1.61)	
LITTLE RELEVANT	11 (50%)	11 (50%)	0.71 (0.10: 4.42)	
NONE	11 (47.83%)	12 (52.17%)	0.78 (0.11: 4.77)	
Collaboration				0.9846
NO	21 (52.5%)	19 (47.5%)	1	
YES	3 (50%)	3 (50%)	1.10 (0.21: 5.79)	
DON’T KNOW	6 (54.55%)	5 (45.45%)	0.93 (0.25: 3.42)	
AISP Usefulness				0.7134
NO	0 (0%)	1 (100%)	1	
YES	28 (53.85%)	24 (46.15%)	0.29 (0: 5.63)	
DON’T KNOW	2 (50%)	2 (50%)	0.33 (0: 10.12)	
AISP Advantage in Care				0.6757
NO	2 (66.67%)	1 (33.33%)	1	
YES	23 (54.76%)	19 (45.24%)	1.38 (0.17: 16.05)	
DON’T KNOW	5 (41.67%)	7 (58.33%)	2.27 (0.23: 30.51)	
Join AISP				0.4025
NO	5 (41.67%)	7 (58.33%)	1	
YES	25 (55.56%)	20 (44.44%)	0.59 (0.16: 2.03)	
Participate in AISP Activities				0.6365
NO	17 (50%)	17 (50%)	1	
YES	13 (56.52%)	10 (43.48%)	0.78 (0.27: 2.21)	
PolandDayUsefulness				0.3722
No or Partial	3 (37.5%)	5 (62.5%)	1	
YES	27 (55.1%)	22 (44.9%)	0.52 (0.11: 2.18)	
Volunteer				0.2516
NO	3 (42.86%)	4 (57.14%)	1	
YES	13 (68.42%)	6 (31.58%)	0.37 (0.06: 2.00)	
DON’T KNOW	14 (45.16%)	17 (54.84%)	0.94 (0.18: 4.51)	
Research				0.5067
YES	29 (51.79%)	27 (48.21%)	1	
DON’T KNOW	1 (100%)	0 (0%)	0.36 (0.00: 7.00)	
Genetic Donation				0.2339
YES	26 (50%)	26 (50%)	1	
DON’T KNOW	4 (80%)	1 (20%)	0.33 (0.03: 1.96)	
Family Involvement in Genetic Research				0.5371
NO	1 (25%)	3 (75%)	1	
YES	23 (53.49%)	20 (46.51%)	0.37 (0.03: 2.50)	
DON’T KNOW	6 (60%)	4 (40%)	0.3 (0.02: 2.58)	

Univariate logistic regression analysis was carried out on the complete set of data and is shown in Table 3. Multivariate analysis (Table 4) confirmed a statistically significant effect regarding time to diagnosis confirmation, the region of Italy where patients live and having to forgo visits and treatments, and on the possibility of receiving the diagnosis out of the region of origin (*p*-values: 0.0350, 0.0075 and 0.0016, respectively). In particular, the chance of having the diagnosis out of the region of origin was about 4 times more likely in patients who had a “late confirmed diagnosis” than that in patients with an “early confirmed diagnosis” (OR (95%C.I.) = 4.10 (1.10 : 18.05)). Patients not living in Liguria or Lombardia were about 8 times more likely to be diagnosed out of their own region than patients living in Liguria or Lombardia (OR (95%C.I.) = 8.17 (1.69: 60.72)). Moreover, taking patients that answered “no” to the “Forgo Treatment” question as the reference group, the chance of being diagnosed out of the region of origin was about 12 times higher than in patients that had to give up the necessary care (OR (95%C.I.) =11.59 (2.46: 84.32)).

**Table 4:** Multivariate analysis, the predictor effects on the diagnosis region (*N* = 57). Results are expressed as odds ratio (OR) with 95% confidence interval (95%CI); *p*-value: Likelihood Ratio *p*-value

Characteristic	OR (95%C.I.)	*p*-value
(Intercept)	0.04 (0: 0.25)	0.0001
Time of Diagnosis		0.0350
EARLY	1	
LATE	4.10 (1.10: 18.05)	
Macro Region		0.0075
LIGURIA, LOMBARDIA		
OTHERS	8.17 (1.69: 60.72)	
Forgo Treatment		0.0016
NO	1	
YES	11.59 (2.46: 84.32)	
DON’T KNOW	0.54 (0.07: 3.12)	

### Time needed for diagnosis

**Fig. 1:**
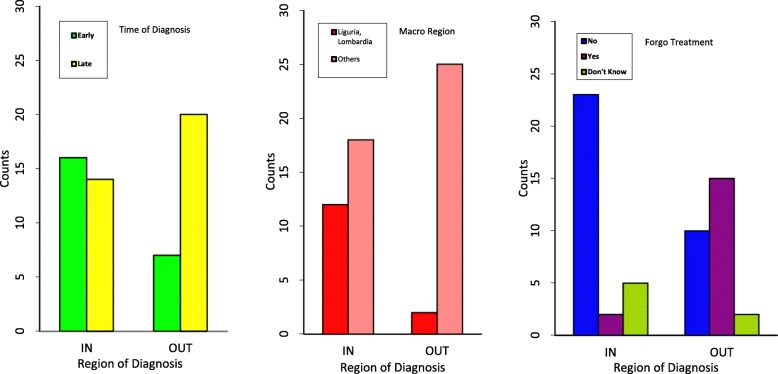
Bar-Plot of the Time of Diagnosis, Macro Region and Forgo Treatment in the Region of Diagnosis

Table 5 reports the descriptive statistics of demographic and clinical factors related to the time needed for diagnosis. With regard to early diagnoses (within the first year of life), they were carried out in neonatology centers in 43.47% of cases. Specialized centers for rare diseases provided an early diagnosis in a further 30.43% of cases. On the other hand, with regard to late diagnoses, they were mainly carried out (44.12%) in specialized centres for rare diseases (*p*-value = 0.0015). Moreover, 76.92% of patients receiving a late diagnosis reported difficulties in finding a specialist to take care of them, while only 23.08% of patients receiving early diagnosis reported this sort of difficulty (*p*-value = 0.0193). Univariate logistic regression analysis carried out on the complete set of data is shown in Table 5. Multivariate analysis (Table 6) confirmed the statistically significant effect of difficulty in identifying a specialist for treatments, having received the diagnosis inside or outside the region of origin and AISP’s role in promoting relationships with health services and operators on time needed for confirmed diagnosis (*p*-values: 0.0154, 0.0497 and 0.0114, respectively). Specifically, the probability of a delayed diagnosis was 5 times higher in patients who had difficulty in identifying a specialist who could provide treatment (OR (95%C.I.) =5.23 (1.48: 21.25)). Moreover, a late PS diagnosis was 3.5 times more likely in patients who were diagnosed outside their region of origin than in those diagnosed inside their region, (OR (95%C.I.) =3.54 (1.01: 14.27)). Finally, a delay in diagnosis was about 94% less likely in patients who considered medical days dedicated to PS useful than in the others (OR (95%C.I.) = 0.06 (0.00: 0.58)) (Fig. 1).

**Table 5:** Contingency tables and summary output of the univariate analysis. Characteristic: variable taken into account; OR (95% CI): Odds Ratio with 95% Confidence Interval; *p*-value: Likelihood Ratio *p*-value. ^a^Variables entering the multivariate analysis (see the text for abbreviations and further details)

Characteristic	Descriptive statistics		Univariate analysis	
	Time of Diagnosis		OR (95%C.I.)	*p*-value
	EARLY	LATE		
Gender				0.1182
MALE	13 (52%)	12 (48%)	1	
FEMALE	10 (31.25%)	22 (68.75%)	2.31 (0.81: 6.86)	
Macro Region				0.7131
LIGURIA, LOMBARDIA	5 (35.71%)	9 (64.29%)	1	
OTHERS	18 (41.86%)	25 (58.14%)	0.8 (0.23: 2.62)	
Region of Diagnosis^a^				0.0372
IN	16 (53.33%)	14 (46.67%)	1	
OUT	7 (25.93%)	20 (74.07%)	3.11 (1.07: 9.7)	
Who Diagnosed^a^				0.0015
NEONATOLOGY UNIT	10 (100%)	0 (0%)	1	
PEDIATRICIAN	2 (50%)	2 (50%)	21 (1.22: 3297.07)	
GENERAL PRACTITIONER	0 (0%)	1 (100%)	63 (1.55: 22813.53)	
SPECIALIZED MED CENTRE	7 (31.82%)	15 (68.18%)	43.4 (4.52: 5865.49)	
SELF-DIAGNOSIS	1 (14.29%)	6 (85.71%)	91 (6.1: 14644.97)	
OTHER	3 (23.08%)	10 (76.92%)	63 (5.62: 8933.3)	
Time of Symptoms Onset				0.3965
AT BIRTH	22 (42.31%)	30 (57.69%)	1	
LATER	1 (20%)	4 (80%)	2.21 (0.37: 23.2)	
Difficulty Finding a Specialist for Treatment^a^				0.0193
NO	17 (58.62%)	12 (41.38%)	1	
YES	6 (23.08%)	20 (76.92%)	4.42 (1.46: 14.66)	
DON’T KNOW	0 (0%)	2 (100%)	7 (0.51: 998.96)	
Quality of Treatment				0.5070
EXCELLENT	7 (43.75%)	9 (56.25%)	1	
GOOD	13 (50%)	13 (50%)	0.79 (0.23: 2.67)	
SUFFICIENT	2 (28.57%)	5 (71.43%)	1.74 (0.31: 12)	
UNSATISFACTORY	1 (16.67%)	5 (83.33%)	2.89 (0.44: 32.89)	
OTHER	0 (0%)	2 (100%)	3.95 (0.27: 577.44)	
Impact^a^				0.0160
NOT MY CASE	15 (62.5%)	9 (37.5%)	1	
NONE	4 (20%)	16 (80%)	5.98 (1.7: 24.58)	
IMPORTANT PSY IMPACT	2 (18.18%)	9 (81.82%)	6.2 (1.38: 38.2)	
MILD PSY IMPACT	1 (100%)	0 (0%)	0.54 (0: 11.32)	
FUNCTIONAL IMPAIRMENT	1 (100%)	0 (0%)	0.54 (0: 11.32)	
Reference Figure during Treatment				0.8297
NO	6 (37.5%)	10 (62.5%)	1	
YES	13 (39.39%)	20 (60.61%)	0.94 (0.27: 3.08)	
DON’T KNOW	4 (50%)	4 (50%)	0.62 (0.12: 3.22)	
Feeling Understood during Treatment				0.5698
NO	3 (30%)	7 (70%)	1	
YES	17 (40.48%)	25 (59.52%)	0.68 (0.15: 2.65)	
DON’T KNOW	3 (60%)	2 (40%)	0.33 (0.04: 2.54)	
Knowledge of tax Exemption				0.2009
NO	0 (0%)	3 (100%)	1	
YES	23 (42.59%)	31 (57.41%)	0.19 (0: 2.12)	
How They Found Out about Exemption^a^				0.0468
GENERAL PRACTITIONER	7 (87.5%)	1 (12.5%)	1	
REGION BOOKING MEDICAL CENTER	10 (35.71%)	18 (64.29%)	8.81 (1.6: 92.23)	
AISP	4 (28.57%)	10 (71.43%)	11.67 (1.78: 137.63)	
REFERENCE MEDICAL CENTER	2 (50%)	2 (50%)	5 (0.45: 80.16)	
Exemption				0.1047
NO	2 (18.18%)	9 (81.82%)	1	
YES	21 (45.65%)	25 (54.35%)	0.31 (0.06: 1.26)	
Sufficiency of the Exemption				0.1556
NO	3 (25%)	9 (75%)	1	
YES	14 (50%)	14 (50%)	0.37 (0.08: 1.45)	
Feeling of Protection				0.1048
NO	8 (29.63%)	19 (70.37%)	1	
YES	8 (66.67%)	4 (33.33%)	0.23 (0.05: 0.9)	
DON’T KNOW	7 (38.89%)	11 (61.11%)	0.67 (0.19: 2.3)	
Forgo Treatment				0.3773
NO	16 (48.48%)	17 (51.52%)	1	
YES	5 (29.41%)	12 (70.59%)	2.14 (0.66: 7.62)	
DON’T KNOW	2 (28.57%)	5 (71.43%)	2.07 (0.43: 12.88)	
General Practitioner _Pediatrician ‘s Role				0.2250
FUNDAMENTAL	4 (80%)	1 (20%)	1	
QUITE RELEVANT	2 (28.57%)	5 (71.43%)	6.6 (0.71: 99.65)	
LITTLE RELEVANT	10 (45.45%)	12 (54.55%)	3.57 (0.54: 39.93)	
NONE	7 (30.43%)	16 (69.57%)	6.6 (1: 74.93)	
Collaboration				0.2571
NO	18 (45%)	22 (55%)	1	
YES	3 (50%)	3 (50%)	0.82 (0.16: 4.32)	
DON’T KNOW	2 (18.18%)	9 (81.82%)	3.12 (0.76: 17.93)	
AISP Usefulness				0.767
NO	0 (0%)	1 (100%)	1	
YES	22 (42.31%)	30 (57.69%)	0.45 (0: 8.88)	
DON’T KNOW	1 (25%)	3 (75%)	0.78 (0: 28.48)	
AISP Advantage in Care				0.1429
NO	2 (66.67%)	1 (33.33%)	1	
YES	19 (45.24%)	23 (54.76%)	2.01 (0.25: 23.32)	
DON’T KNOW	2 (16.67%)	10 (83.33%)	7 (0.65: 109.9)	
Join AISP				0.2438
NO	3 (25%)	9 (75%)	1	
YES	20 (44.44%)	25 (55.56%)	0.46 (0.1: 1.67)	
Participate in AISP Activities^a^				0.0103
NO	9 (26.47%)	25 (73.53%)	1	
YES	14 (60.87%)	9 (39.13%)	0.24 (0.08: 0.72)	
PolandDay Usefulness^a^				0.0095
No or Partial	0 (0%)	8 (100%)	1	
YES	23 (46.94%)	26 (53.06%)	0.07 (0: 0.58)	
Volunteer				0.4363
NO	2 (28.57%)	5 (71.43%)	1	
YES	6 (31.58%)	13 (68.42%)	0.94 (0.14: 5.3)	
DON’T KNOW	15 (48.39%)	16 (51.61%)	0.48 (0.08: 2.36)	
Research				0.6361
YES	23 (41.07%)	33 (58.93%)	1	
DON’T KNOW	0 (0%)	1 (100%)	2.1 (0.11: 312.63)	
Genetic Donation				0.0715
YES	19 (36.54%)	33 (63.46%)	1	
DON’T KNOW	4 (80%)	1 (20%)	0.19 (0.02: 1.15)	
Family Involvement in Genetic Research				0.3269
NO	2 (50%)	2 (50%)	1	
YES	15 (34.88%)	28 (65.12%)	1.84 (0.26: 13.04)	
DON’T KNOW	6 (60%)	4 (40%)	0.69 (0.08: 6.17)

**Table 6:** Multivariate analysis, the predictor effects on the time needed for diagnosis (*N* = 57). Results are expressed as odds ratio (OR) with 95% confidence interval (95%CI); *p*-value: Likelihood Ratio *p*-value

Characteristic	OR (95%C.I.)	*p*-value
(Intercept)	4.35 (0.4: 594.1)	0.2624
Difficulty Finding a Specialist for Treatment		0.0154
NO	1	
YES	5.23 (1.48: 21.25)	
DON’T KNOW	13.45 (0.81: 2064.32)	0.0497
Region of Diagnosis		
IN	1	
OUT	3.54 (1.01: 14.27)	
PolandDay Usefulness		0.0114
NO OR PARTIAL	1	
YES	0.06 (0: 0.58)

## Discussion

According to the World Health Organization (WHO) Constitution, “the enjoyment of the highest attainable standard of health is one of the fundamental rights of every human being”. However, the decades-long diagnostic odysseys for many rare diseases patients has scarcely been studied [19]: guaranteeing that all people living with a rare disease may receive an accurate and prompt diagnosis is generally agreed upon as being an urgent need [20].

Previous studies have reported that available treatments and care for rare diseases are varied and heterogeneous, reflecting political, economic and geographic differences among countries [21]. The health care system in Italy is a regionally based national health service known as Servizio Sanitario Nazionale (SSN). While the national health service ensures that the general objectives and fundamental principles of the national health care system are met, the regional governments in Italy are responsible for ensuring the delivery of a benefits package to the population. They are autonomous and are responsible for organizing networks for patients with rare diseases [22].

The main aims of our study were to assess the potential link between the geographic origin of patients and time spent before obtaining a PS diagnosis, and to evaluate the quality of the received treatments. Data on patients’ experiences of diagnostic path reported in our research show a lower diagnostic threshold than those reported in the survey carried out by the European Organisation for Rare Diseases (Eurordis) on eight heterogeneous rare diseases in 2007 [23]. In particular, Italian patients with PS had to travel to a location outside of their home region to obtain the diagnosis almost twice as often as European rare diseases patients. We can assume that the diagnostic odyssey of Italian patients with PS is not only due to the lack of adequately trained healthcare professionals and dedicated health facilities in the area where patients affected by PS are born, but also to the the lack of knowledge about the services that are available for PS patients by health professionals even in regions where an official PDTA has already been released. Although appropriate clinical facilities are already in place, diagnosis can still be delayed, thus confirming the limited awareness by the general population.

Our data show that receiving a correct diagnosis in one’s own region is essential for the therapeutic continuity, which means that the patient can be appropriately followed-up by the regional health system. Italian patients affected by PS have reported similar experiences to those identified internationally among people living with rare diseases, their caregivers and families [24]. Failure to obtain a correct diagnosis is an onerous experience for patients and their families as they are forced to consult numerous physicians and to undergo many, often expensive, consultations and clinical tests, a situation which causes psychological distress and consequently, additional stress [25]. Furthermore, without a diagnosis, the patient’s medical or social needs may not receive adequate attention thereby creating the feeling of being “invisible” [26]. Taken together, these aspects affect the responsible participation by the patient in the therapeutic path that represents an important factor both for adults and for minors, which was recently enhanced in Italy by specific legislation [27, 28].

The positive correlation between logistic regression analysis and the patient’s engagement in AISP (which includes participation in social activities as well as access to a dedicated psychological support plan and to consultation with physicians specifically trained in PS) is absolutely in line with the litterature [29] and can be explained by items that include confidence and empathy, which may be pivotal in influencing subjective well-being rating more than any other clinical symptom of PS.

Our finding of a significant correlation between the patients’ decision to forgo follow-up healthcare programs and the patients’ region of origin corroborates the results of previous studies on rare diseases [30]. Accordingly, the physical distance from high-quality health services may directly impact upon the patient’s social, psychological, and occupational functioning. The ethical principles of non-discrimination, justice, equity and solidarity require adequate consideration, as does the protection of the needs of individuals with rare diseases and their families [31]. Taken together, the paucity of specific health policies for rare diseases, the scarcity of skills, the lack of a national support plan and of specific training programs result in delayed diagnoses, difficulties in accessing care, inadequate treatment, increased physical discomfort, socio-psychological issues for patients and their families and, finally, in a loss of confidence in the public health system [32]. The all too frequent difficulty in finding a specialist who can confirm the diagnosis, regardless of the patient’s region of origin, may partly be ascribed to the absence of a specific national plan and to the lack of exchange of knowledge and information within a structured and coordinated network. In line with previous studies, we agree that the lack of adequate information and training programs, as well as of a national medical network able to offer consultancy support, leaves the general practitioner with a marginal role and increases the number of self-diagnoses [24]. As already reported, drawing up multidisciplinary integrated paths is crucial in order to allow medical staff to share care protocols based on best practices and to ensure adequate and effective treatment for patients with a rare disease [33].

As confirmed by this study, patient advocacy organizations (PAOs) currently play a key role in the management of patients seeking a clinical reference, thereby improving the assistance and the quality of life of people living with a rare disease [34, 35]. Our study was designed with the cooperation of AISP to identify the main issues concerning the health services available to Italian patients with Poland syndrome. Based on the analysis of our data, in fact, AISP then took targeted actions to strengthen care services in southern and central Italy by organizing two informative clinical events in Puglia (“The network for care and research” - San Giovanni Rotondo (Foggia), 25th November, 2017) and in Emilia Romagna (“Open Day on Poland Syndrome” – Modena, 13th October, 2018). Involving health facilities which have all the medical specializations that are needed to support the affected patients eventually led to the birth of two new centres of reference for the diagnosis and treatment of patients with Poland Syndrome.

When interpreting our data, we have to consider some limitations. First of all, only patients with an overall good attitude towards being surveyed took part in the study and filled in the questionnaire exhaustively. Moreover, the sample size is rather small and does not include patients from all Italian regions. Furthermore, given the heterogeneity of the patients’ age, questionnaires were filled in by caregivers in case of minors, while adult patients filled in the questionnaire autonomously. Differences between self- and proxy-completed responses on health status surveys are well described in the literature [36, 37]. For example, some studies have reported that parents tend to assign a lower score on health-related quality of life questions regarding their children with a chronic health condition [38–40]. However, the accuracy of the responses is strictly linked to the degree of confidence the proxy respondent has with the presented issues. In our study, the topics we investigated mainly concern objective questions whose answers are probably best known by the patients’ parents. Lastly, we did not thoroughly assess the real reasons for forgoing treatment by patients who had previously been receiving appropriate support. A further extension of this study based on qualitative interviews with patients, clinicians and other stakeholders (such as AISP members) will allow us to acquire more details and explanations of the phenomenon and will re-evaluate health services for Italian patients suffering from Poland syndrome taking into account the activity of the two new strategically located Italian reference centers.

## Conclusions

This study underlines that the healthcare needs of people living with PS are not being fully met in Italy. Our analysis of patients’ experiences highlights many gaps and a lack of homogeneity in the diagnostic and therapeutic path of PS patients in our country. The implementation of an effective information network to promote research, stimulate clinical collaboration and improve the quality of life of people with rare diseases in general is, to date, predominantly supported by PAOs.

Adequate empowerment of PAOs within the National Health Service and the institutional bodies where health policies are defined would allow these volunteer associations to evolve from merely being a voice of the shortcomings of the public sector to a key player within the territory; this synergy would thus result in a greater application of the principle of subsidiarity.

## Data Availability

The datasets used and/or analyzed during the current study are available from the corresponding author on reasonable request.
